# Brushing Up on Cartilage Lubrication: Polyelectrolyte-Enhanced Tribological Rehydration

**DOI:** 10.1021/acs.langmuir.4c00598

**Published:** 2024-05-07

**Authors:** Robert J. Elkington, Richard M. Hall, Andrew R. Beadling, Hemant Pandit, Michael G. Bryant

**Affiliations:** † Institute of Functional Surfaces, Mechanical Engineering, University of Leeds, Leeds LS2 9JT, Yorkshire, U.K.; ‡ School of Engineering College of Engineering and Physical Sciences, University of Birmingham, Birmingham B15 2TT, West Midlands, U.K.; § Leeds Institute of Rheumatic and Musculoskeletal Medicine, Chapel Allerton Hospital, Chapeltown Road, Leeds LS7 4SA, Yorkshire, U.K.

## Abstract

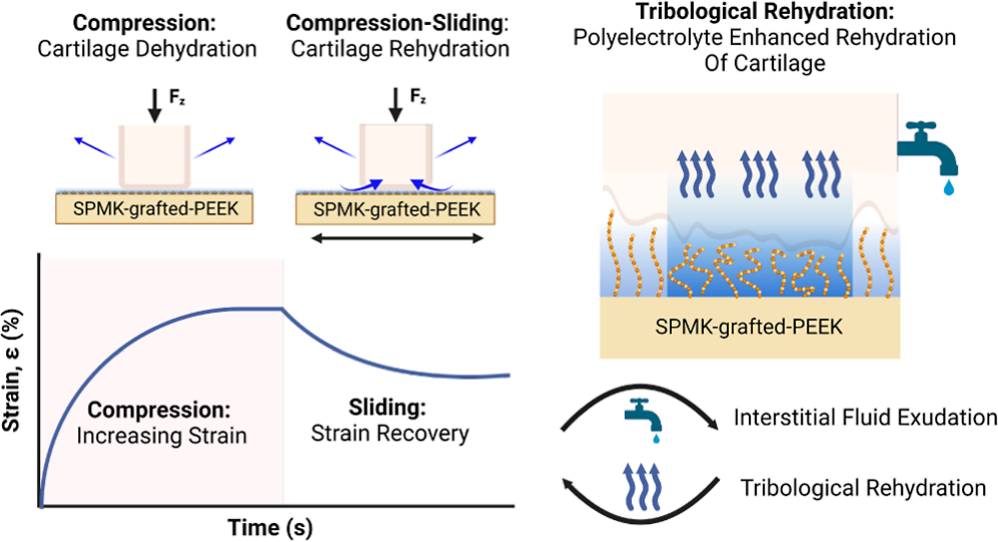

This study presents new insights into the potential role of polyelectrolyte interfaces in regulating low friction and interstitial fluid pressurization of cartilage. Polymer brushes composed of hydrophilic 3-sulfopropyl methacrylate potassium salt (SPMK) tethered to a PEEK substrate (SPMK-*g*-PEEK) are a compelling biomimetic solution for interfacing with cartilage, inspired by the natural lubricating biopolyelectrolyte constituents of synovial fluid. These SPMK-*g*-PEEK surfaces exhibit a hydrated compliant layer approximately 5 μm thick, demonstrating the ability to maintain low friction coefficients (μ ∼ 0.01) across a wide speed range (0.1–200 mm/s) under physiological loads (0.75–1.2 MPa). A novel polyelectrolyte-enhanced tribological rehydration mechanism is elucidated, capable of recovering up to ∼12% cartilage strain and subsequently facilitating cartilage interstitial fluid recovery, under loads ranging from 0.25 to 2.21 MPa. This is attributed to the combined effects of fluid confinement within the contact gap and the enhanced elastohydrodynamic behavior of polymer brushes. Contrary to conventional theories that emphasize interstitial fluid pressurization in regulating cartilage lubrication, this work demonstrates that SPMK-*g*-PEEK’s frictional behavior with cartilage is independent of these factors and provides unabating aqueous lubrication. Polyelectrolyte-enhanced tribological rehydration can occur within a static contact area and operates independently of known mechanisms of cartilage interstitial fluid recovery established for converging or migrating cartilage contacts. These findings challenge existing paradigms, proposing a novel polyelectrolyte–cartilage tribological mechanism not exclusively reliant on interstitial fluid pressurization or cartilage contact geometry. The implications of this research extend to a broader understanding of synovial joint lubrication, offering insights into the development of joint replacement materials that more accurately replicate the natural functionality of cartilage.

## Introduction

Articular cartilage is a highly specialized avascular connective tissue of mammalian diarthrodial joints, approximately 2–4 mm thick in human hip (acetabular-femoral) and knee (tibiofemoral) joints.^1^ Within synovial joints, cartilage provides extremely low friction coefficients, below 0.01, and withstands high pressures up to 10–20 MPa across an 80 year lifespan.^2,3^ Comprising roughly a 20% collagen matrix and 80% water, cartilage’s avascular nature limits its healing capacity after trauma or osteoarthritis onset.^4^ As a result, the prevalence of surgical interventions for joint repair (total joint arthroplasty) is expected to surge, with projections indicating a doubling of patient demand in OECD countries by 2050.^5,6^ This trend presents a considerable challenge to global health systems.^6,7^ This is particularly concerning for younger patients, who face higher risks of early prosthesis failure and subsequent complex revision surgeries.^8,9^ Alternative conservative treatments, such as focal cartilage repair or hemiarthroplasty using hard engineering biomaterials like cobalt–chromium–molybdenum (CoCrMo), frequently result in higher revision rates due to excessive wear of cartilage.^10−12^ These approaches fail to replicate cartilage’s unique multimodal lubrication and fluid load support, crucial for protecting the collagen matrix.^13,14^ Focal repair of osteochondral lesions remains an ongoing clinical challenge in orthopedics.^15^ Present tissue engineering approaches fail to replicate the structural properties of cartilage leading to inconsistent patient outcomes,^16^ requiring further development of materials that can emulate and support the native tribology of articular cartilage.^17^

**Figure 1:**
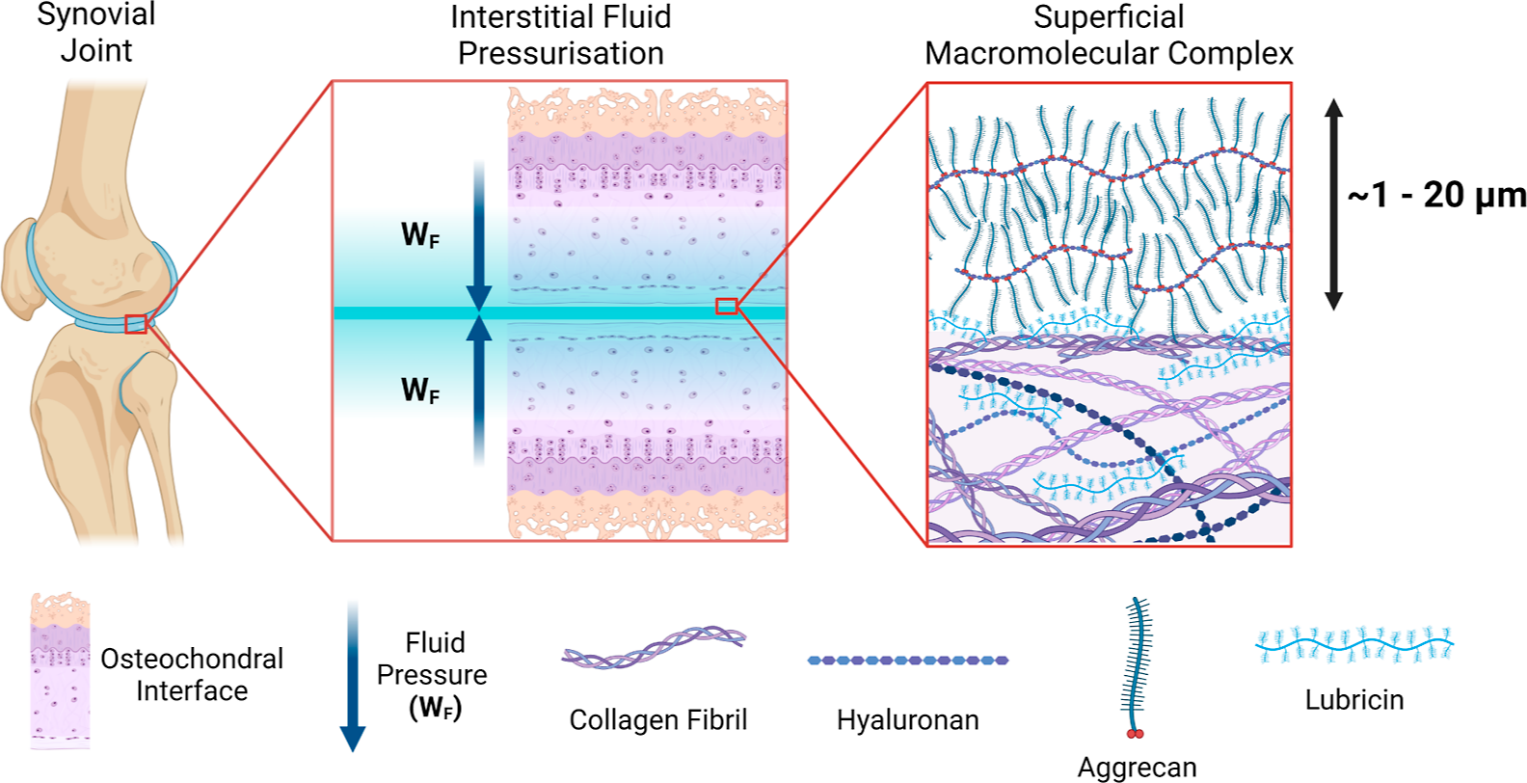
Schematic showing the multiscale tribological function of cartilage. **Synovial joint:** covered with approximately 4 mm of articular cartilage.^1^**Interstitial fluid pressurization:** upward of 90% of an articular joint’s load is borne over interstitial fluid pressure.^14,19^**Superficial macromolecular complex**: ∼1–20 μm-thick gel-like complex of hyaluronic acid, proteoglycan aggregates (hyaluronan-aggrecan), and lubricin (proteoglycan-4) adsorbed on the superficial osteochondral surface.^3,22,23^

Synovial lubrication is highly responsive, modulating a high degree of fluid pressurization and low coefficient of friction (CoF, μ) through complex interactions between surface-bound macromolecules, regulation of interstitial fluid flow due to poroviscoelastic biomechanics, and elastohydrodynamic lubrication borne through the congruence of articulating surfaces.^3,13^Figure 1 shows the multiscale tribological function of cartilage. It has been mooted that interstitial fluid pressurization can support upward of 90% of the joint load.^18^ Experimental and theoretical evidence for the role of cartilage interstitial fluid pressurization demonstrates the equilibrium CoF during sliding conditions, denoted as μ_eq_, is a function of the friction from the solid phase (μ_s_) and the ratio of interstitial fluid load to the normal force , as described by references 14 and 19–21.^14,−^ This relationship demonstrates that for cartilage CoF to remain low, a high degree of fluid load support [*W*_f_(*t*)] must be maintained, which can be inferred from a reduced cartilage strain [ϵ(*t*)] as a ratio of strain with zero interstitial pressure (ϵ_0_),^14,21^ whereas boundary lubrication is expected to occur where pressurization subsides and solid contact occurs with cartilage.^3,19^ This aqueous lubrication boundary layer is an approximately ∼1–20 μm-thick gel-like macromolecular complex adsorbed on the superficial surface of articular cartilage,^22−26^ composed of specialized molecules in the synovial fluid including hyaluronic acid, proteoglycan aggregates (hyaluronan-aggrecan), and lubricin (proteoglycan-4), each characterized by large hydrophilic charged domains with a high water carrying capacity.^3,23^ At the nanoscale level of cartilage tribology, sliding between confined hydration shells leads to a mode of hydration lubrication exhibiting CoF < 0.001, as shear forces are dissipated through rapid exchange of water molecules between adjacent shells.^2,27−29^
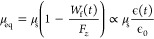
1

Human synovial joints typically experience spatially averaged and peak loads of between 0.75 and 20 MPa during gait and activity^2,30,31^ and for sustained lifetime function are required to recuperate interstitial fluid that is exuded during loading (eq 1). *In vivo* studies of tibiofemoral cartilage have measured strain (ϵ) across a range of activities including half bodyweight static loading (ϵ ∼ 12%);^32^ gait (ϵ ∼ 7–23%);^33^ 10 min after jogging (ϵ ∼ 4%);^34^ and knee bending (ϵ ∼ 3–8%).^35^ Cartilage has been shown to recover interstitial fluid due to passive free swelling driven by the viscoelastic recovery of cartilage when unloaded in a supine position^34^ and also during physical activity between intermittent periodic loading as the cartilage contact area migrates.^36,37^ The latter is akin to experimental observations of a migrating contact area (MCA) which can maintain low CoF < 0.02 and modulate high levels of interstitial fluid pressurization as the contact migrates.^38,39^ Recent advances by Burris and Moore and Burris^40^ have introduced a novel mechanism termed *tribological rehydration* for cartilage interstitial fluid recovery. This process occurs when sliding convex cartilage plugs with convergent stationary contact areas (cSCA) generate a wedge effect at the contact inlet, producing sufficient hydrodynamic pressures to drive fluid recovery exhibited as a reduction of strain due to augmentation of cartilage interstitial fluid pressurization.^40,41^

Assemblies of polymer chains tethered at one end onto a substrate, *polymer brushes*, have attracted significant attention as potential cartilage replacement materials and as tunable biomimetic polyelectrolytes to explore superficial macromolecular complexes.^3,31,42−48^ Polymer brush systems can provide a stable aqueous lubrication interface with CoF as low as 0.001 at physiological loads of up to 10 MPa,^2,3,27,47^ owing to their high affinity for water and brush-like structure resisting deformation through electrostatic and osmotic repulsion.^3,47^ Due to their biocompatibility, tunable mechanical properties, superior lubrication, hydration control, and chemical functionalization possibilities, various orthopedic applications of polymer brushes have been explored, including as viscosupplementation;^49,50^ direct attachment to cartilage; and^51,52^ bioinspired lubricious surface coating for acetabular hip replacement surfaces^53−55^ and as brush-terminated hydrogels designed to mimic cartilage.^44,56,57^ Polymer brushes comprised of poly(2-methacryloyloxyethyl phosphorylcholine) (MPC) attached to CoCrMo surfaces have demonstrated potential as cartilage-mimetic interfaces, exhibiting physiological coefficients of friction (μ < 0.01) and reduced collagen degradation relative to unmodified surfaces across a limited data set of 100 reciprocating cycles.^58^ Notably, investigations of this nature are scarce in the literature.

Recently, polymer brush-functionalized surfaces consisting of 3-sulfopropyl methacrylate potassium salt (SPMK) grafted onto a PEEK substrate (SPMK-*g*-PEEK) have been developed to provide sustained low friction coefficients of <0.02 on cartilage at physiological loads (0.75 MPa).^31,42,59^ SPMK-*g*-PEEK surfaces also possess a unique ability to halve overall cartilage strain throughout 2.5 h of sliding, empirically demonstrated through the use of a flat SCA cartilage, specifically designed to negate any contributions from known mechanisms of MCA or cSCA interstitial fluid recovery.^14,31^ Such findings indicate a novel mechanism of enhanced rehydration attributable to the SPMK polyelectrolyte interface.^31^ While the sustained low friction can be ascribed to tethered hydrophilic polyelectrolytes providing a high degree of solvent confinement in the contact to provide an effective boundary lubrication layer,^3,27,31,46^ the role of polyelectrolyte-enhanced tribological rehydration remains poorly understood.

The primary objective of this study is to deepen the understanding of cartilage–polyelectrolyte tribology by investigating the role of polymer brushes in facilitating tribological rehydration.^31^ This research hypothesizes that cartilage–polyelectrolyte interfaces, characterized by surface-grafted polyelectrolytes that maintain hydration under mechanical load,^3,27^ exhibit high compliance,^47^ and increase aqueous film thickness,^60−62^ may generate elevated fluid pressures at the interface. Such pressures are theorized to support the recovery of cartilage interstitial fluid.^31,42^ This supposition underpins our examination of friction and tribological rehydration within a hydrodynamic framework, necessitating a detailed tribological analysis of cartilage–SPMK interfaces across various speed and load conditions. Specifically, this study seeks to identify and quantify the critical speed and load parameters that facilitate observable strain recovery in cartilage interfaced with SPMK-*g*-PEEK, thereby providing evidence of tribological rehydration. Second, this study aims to explore the current mechanistic arithmetic^41^ and empirical models^40^ for tribological rehydration alongside interstitial fluid pressurization to form a hypothesis on the mechanism of polyelectrolyte-enhanced tribological rehydration. Experiments will use a SCA cartilage contact, for which no demonstration of tribological rehydration exists.^14^ This seeks not only to elucidate the underlying principles of polyelectrolyte-enhanced tribological rehydration but also to contribute to the development of functional biomimetic cartilage repair materials and deeper insights into the potential mechanisms of adsorbed biopolyelectrolytes within the superficial macromolecular complex.

## Methods

### Sample Preparation

#### Materials

PEEK 450G (Victrex, UK) sheets (5 mm thick, cut into 25 × 25 mm squares) were sourced from RS Components, UK. These were polished using a graded series of abrasive papers and suspensions to achieve an arithmetic surface roughness *R*_a_ ∼ 100 nm, confirmed with a Talysurf PGI NOVUS profilometer. The SPMK monomer (>98% purity) and phosphate-buffered saline (PBS) tablets were obtained from Sigma-Aldrich, UK, and used as received. For all experiments, SPMK-*g*-PEEK interfaces are explored in the context of biomedical implant materials and hence in an isotonic PBS environment to mimic physiological ion concentrations and osmolarity, containing 137 mM sodium chloride (Na^+^Cl^–^), 10 mM phosphate buffer (K^+^), and 2.7 mM potassium chloride (K^+^Cl^–^) with a pH of approximately 7.4.

#### SPMK-*g*-PEEK

To produce SPMK-*g*-PEEK, polished PEEK samples were initially cleaned with acetone and isopropanol, then immersed in a 1 mol L^–1^ solution of SPMK in deionized water. This solution was purged of oxygen. The samples underwent UV photopolymerization in an Analytik Jena UVP Cross-linker CL-3000L at a wavelength of 365 nm for 90 min, amounting to a total UV exposure of 27 J cm^–2^ to initiate a *grafting from* process for synthesizing high density SPMK polymer brushes on the PEEK surface (SPMK-*g*-PEEK).^3^ This one-step photoinitiated radical polymerization utilizes PEEK’s benzophenone units for self-initiation, thus eliminating the need for additional photoinitiators.^63−65^ The resultant SPMK-*g*-PEEK surfaces had a polyelectrolyte thickness of approximately 350 nm^31^ and feature highly hydrophilic anionic sulfonic acid groups, enhancing water retention and lubricity for biotribological applications. Further details on this method and its underlying chemistry are provided in a previous publication.^31^

#### Cartilage Samples

Flat SCA bovine cartilage plugs (⌀ 4.0 mm and ⌀ 7.2 mm diameter) were extracted from the patellofemoral grooves of bovine (age ∼2 years) stifle joints sourced from John Penny & Sons, Leeds, UK. The extraction used a high-speed rotary tool cooled with a steady stream of PBS. Plugs with surface irregularities or a height slope exceeding 0.2 mm were discarded. Samples were cryopreserved at −18 °C and prior to testing were thawed 12 h before being acclimatized to room temperature for 2 h in PBS.

### Surface Analysis

An NPFLEX (Bruker, USA) optical interferometer was used to measure the surface roughness of the polished unfunctionalized PEEK and SPMK-*g*-PEEK samples using a noncontact vertical scanning interferometry method, analyzing surface reflections to create interference fringes at a 50× optical magnification. Three different 250 × 250 μm areas of each sample were scanned using a high-intensity monochromatic green light to enhance reflection and minimize data loss. Optical profilometry data was processed using Bruker Vision64 software to calculate the mean arithmetic roughness (*R*_a_) for each sample area.

A Tescan Amber X plasma focused ion beam-scanning electron microscope (FIB-SEM) was used to measure the hydrated polyelectrolyte height of the SPMK-*g*-PEEK surfaces. SPMK-*g*-PEEK samples were hydrated by submerging in PBS for 10 min before being placed in a Quorum PP3010 cryo-preparation chamber and frozen in slushed nitrogen (∼−210 °C) before being transferred to the SEM under vacuum to prevent ice formation.^66^ The SPMK-*g*-PEEK samples were then sputter-coated with a 20 nm-thick platinum layer before FIB was used to mill a 100 L × 40 W × 100 D μm cross section. Cross-section images were taken using the SEM to identify the SPMK layer which was validated using energy-dispersive X-ray spectroscopy (EDX) to locate sulfonic acid groups. PBS was specifically used to model the hydrated thickness of SPMK-*g*-PEEK in isotonic environments, where the polyelectrolyte layer will be sensitive to the presence of ionic species, partially collapsing the brush structure due to screening out of electrostatic repulsion along with exclusion of water from the brushes.^61,67,68^

A Bruker dimension icon atomic force microscope (AFM) was used to map the elastic modulus of swollen SPMK-*g*-PEEK samples submerged in PBS. Measurements were performed using SAA-SPH-10UM (Bruker AFM Probes, USA) AFM probes due to their low precalibrated 0.25 Nm^–1^ spring constant and large 10 μm probe radius suitable for measuring soft samples in the sub-kPa range. Indentation force measurements were performed in a 16 × 16 grid (256 total) over a 50 × 50 μm area, and two force maps were each performed on two SPMK-*g*-PEEK samples. Each force–displacement curve was performed with a ramp size of 5.0 μm at a 2 μms^–1^ indentation velocity, as used in similar soft contact research.^69,70^ Data was analyzed using a custom Python script to identify the contact displacement at which the probe engaged with the surface indicated by a reduction in noise and subsequently identify the data region that complies with Hertzian contact theory to calculate elastic modulus.^69,71^ The elastic modulus was calculated for only the first 1 μm of indentation to isolate substrate effects.

### Mechanical Testing

**Figure 2:**
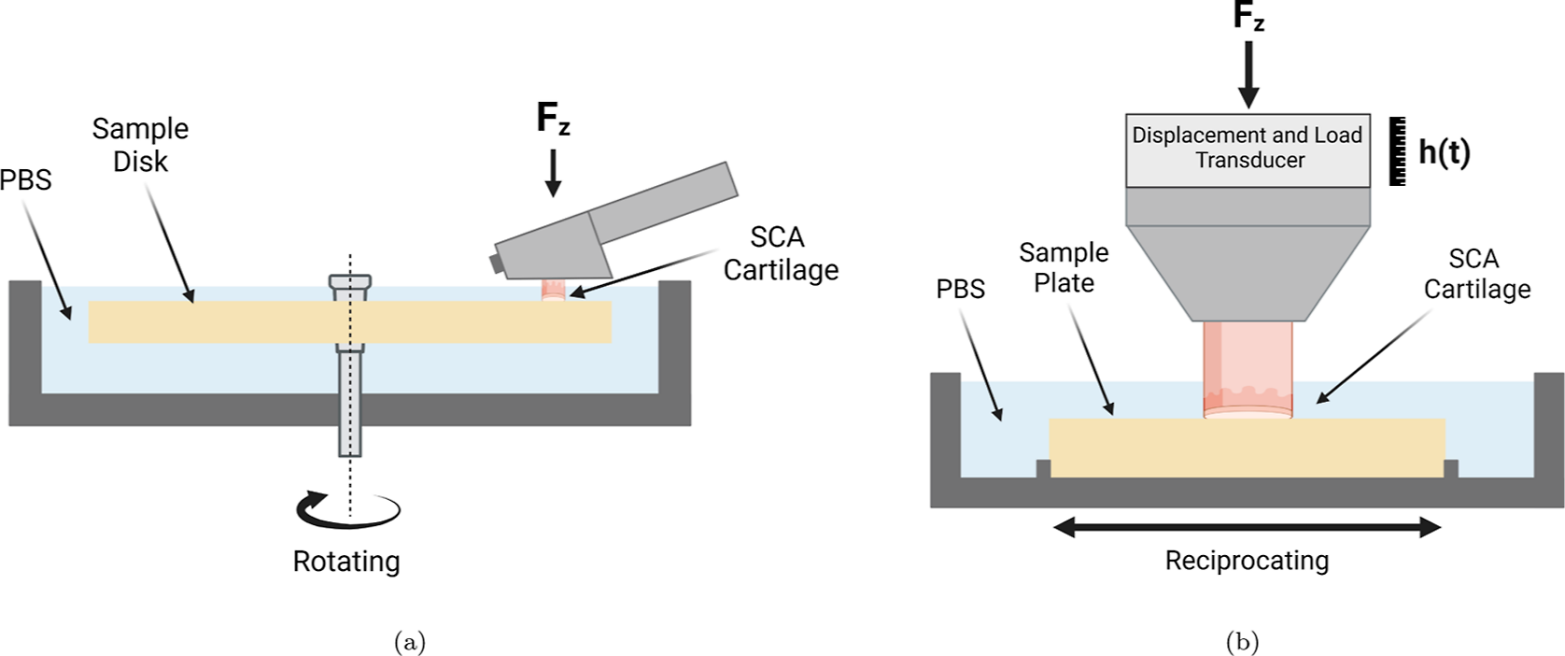
(a) MTM pin-on-disc configuration showing the PEEK/SPMK-*g*-PEEK sample disk submerged in PBS and a ⌀ 4.0 mm SCA cartilage plug mounted in the pin holder. (b) UMT pin-on-plate configuration showing the affixed sample plate in a bath of PBS linearly reciprocating over a distance of 20 mm against a ⌀ 7.2 mm SCA cartilage plug. A constant normal load, *F*_*Z*_, is applied throughout the rehydration cycle, regulated *via* PID control. Additionally, a displacement transducer is employed to record variations in cartilage height [*h*(*t*)] throughout testing.

To understand the tribological behavior of SPMK-*g*-PEEK, speed sweep experiments analogous to Stribeck analysis^72^ were conducted to explore SCA cartilage over a short 5 min loading period, in order to mitigate the effects of rising friction contributions due to the time-dependent loss of interstitial fluid pressurization. Figure 2a shows the pin-on-disk configuration of an MTM (Micro Traction Machine, PCS Instruments, UK) which was used to perform speed sweep analysis of a flat ⌀ 4.0 mm SCA cartilage plug against unfunctionalized PEEK and SPMK-*g*-PEEK disks. Both increasing speed sweeps of 1–200 mm/s and decreasing sweeps of 200–1 mm/s were performed three times for each test condition. A 15 N constant load was applied throughout the test, which for a ⌀ 4.0 mm cartilage plug corresponds to a ∼ 1.2 MPa contact pressure assuming full contact over the SCA contact. Three repeats were performed for each test condition. To ensure a physiological isotonic gradient, all testing was performed fully submerged in PBS. The CoF (μ, eq 2), the ratio of the tangential force (*F*_*X*_) to the applied load (*F*_*Z*_), is recorded throughout the test at a frequency of 1 Hz.
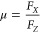
2

Figure 2b shows the UMT TriboLab (Bruker, USA) equipped with a reciprocating linear drive and custom-built lubricant bath used to measure the compression and subsequently strain recovered due to tribological rehydration of a flat ⌀ 7.2 mm SCA cartilage plug sliding against SPMK-*g*-PEEK. Throughout testing, samples remained fully submerged in PBS and closed-loop PID control maintained constant *F*_*Z*_ loading with an accuracy of ±0.5 N and concurrently measured changes in cartilage compression [*h*(*t*)]. The full details of this test setup are described in a previous publication.^31^ A rehydration cycle, lasting 3600 s, was conducted to evaluate the tribological rehydration of SCA cartilage interfacing with SPMK-*g*-PEEK under varying conditions of load and sliding speed. The cycle was divided into two phases: an initial phase of unconfined compression (no sliding) for 1800 s, followed by a 1800 s sliding phase under a constant normal load. The experiments were conducted under three load conditions: *F*_*Z*_ = 10 N, *F*_*Z*_ = 30 N, and *F*_*Z*_ = 90 N which correspond to contact pressures of 0.25, 0.74, and 2.21 MPa, respectively, assuming uniform contact across the cartilage surface, representative of the physiological pressures encountered by tibiofemoral articular cartilage during human gait.^73^ To assess the impact of sliding speed on tribological rehydration, specifically focusing on compression recovery during sliding due to cartilage interstitial fluid recovery, each load condition was tested across a range of speeds (ν) set at 0.1, 0.5, 1, 2, 5, and 10 mm/s each linearly reciprocating across a 20 mm sliding distance. CoF (eq 2) was recorded only for the *F*_*Z*_ = 30 N load condition, as this scenario optimally aligned with the calibrated ranges of tangential load cells available.

Following testing, each cartilage plug was placed in PBS for 1 h to free-swell and recover the compressed height and then stored in a phosphate buffered formalin solution. The uncompressed height of each cartilage plug (*h*_0_) was then measured using a calibrated Keyence VHX-7000 optical microscope with a 20× magnification; the measurement protocol is detailed in a previous publication.^31^ This enabled calculation of the cartilage compression in terms of the overall strain [ε(*t*) = *h*(*t*)/*h*_0_]. Strain recovery (ε_r_), defined in eq 3, was quantified as the difference in total strain observed at the conclusion of the 1800 s compression phase [ε_c_ = ε(*t* = 1800 s)] and the strain measured at the end of the 3600 s sliding phase [ε_s_ = ε(*t* = 3600 s)]. This calculation facilitates a direct comparison of the strain recovery capabilities of the cartilage attributable to tribological rehydration facilitated by the SPMK-*g*-PEEK interface under varying speed and load conditions.
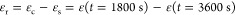
3

## Results

### Surface Analysis

Surface roughness of the unfunctionalized PEEK measured a mean roughness of *R*_a_ = 101 ± 9.8 nm (*N* = 3), and mean roughness of the SPMK-*g*-PEEK measured *R*_a_ = 304 ± 10.9 nm (*N* = 3). The increase in the *R*_a_ value for SPMK-*g*-PEEK indicates that grafting of SPMK has markedly altered the topography of PEEK specifically introducing additional surface features along with increasing the prominence of existing ones. Once hydrated, the SPMK surface features will become obscured as the hydrophilic polymer chains swell to provide an aqueous interface.

**Figure 3:**
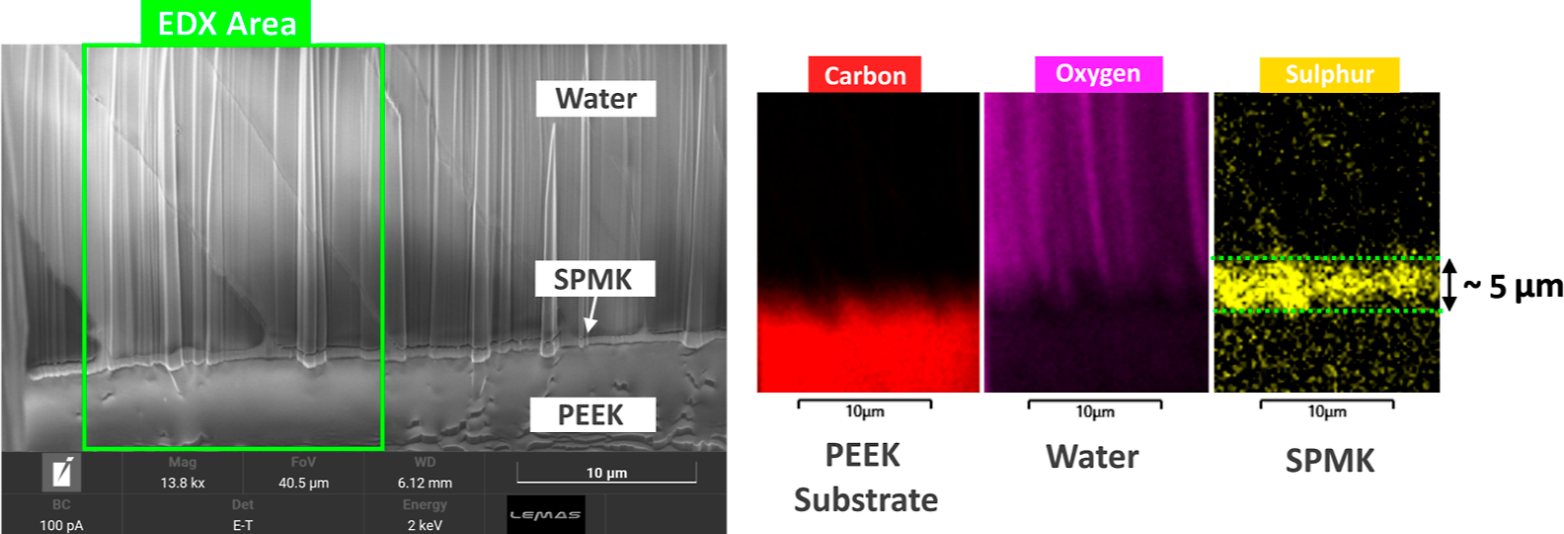
Left: CryoSEM image of swollen SPMK-*g*-PEEK cross section showing the area of EDX analysis. Right: EDX analysis of carbon (red), oxygen (purple), and sulfur (yellow) corresponding to the PEEK substrate, frozen water, and SPMK layers, respectively, measuring a swollen polyelectrolyte layer of approximately 5 μm.

Figure 3 presents the Cryo-FIBSEM imaging and EDX analysis conducted to determine the swollen height of the SPMK layer on the PEEK substrate. SEM imaging alone fails to distinctly delineate the SPMK interlayer, obscured by the presence of frozen water and density variations in the swollen SPMK.^74^ The thickness of the SPMK layer is identified by the region exhibiting the highest sulfur content, attributed to the sulfonic acid groups within the polyelectrolyte layer, with an estimated height of approximately 5 μm. This region lies beneath an oxygen-rich area indicative of frozen water and above a carbon-rich zone representing the PEEK substrate. The spatial resolution limit of EDX composition analysis, typically around 1 μm due to the volumetric interaction of the electron beam,^75^ implies that the measured height of the SPMK layer, while indicative, cannot be precisely quantified through EDX, rendering the derived height as an approximate estimate rather than an exact measurement.

**Figure 4:**
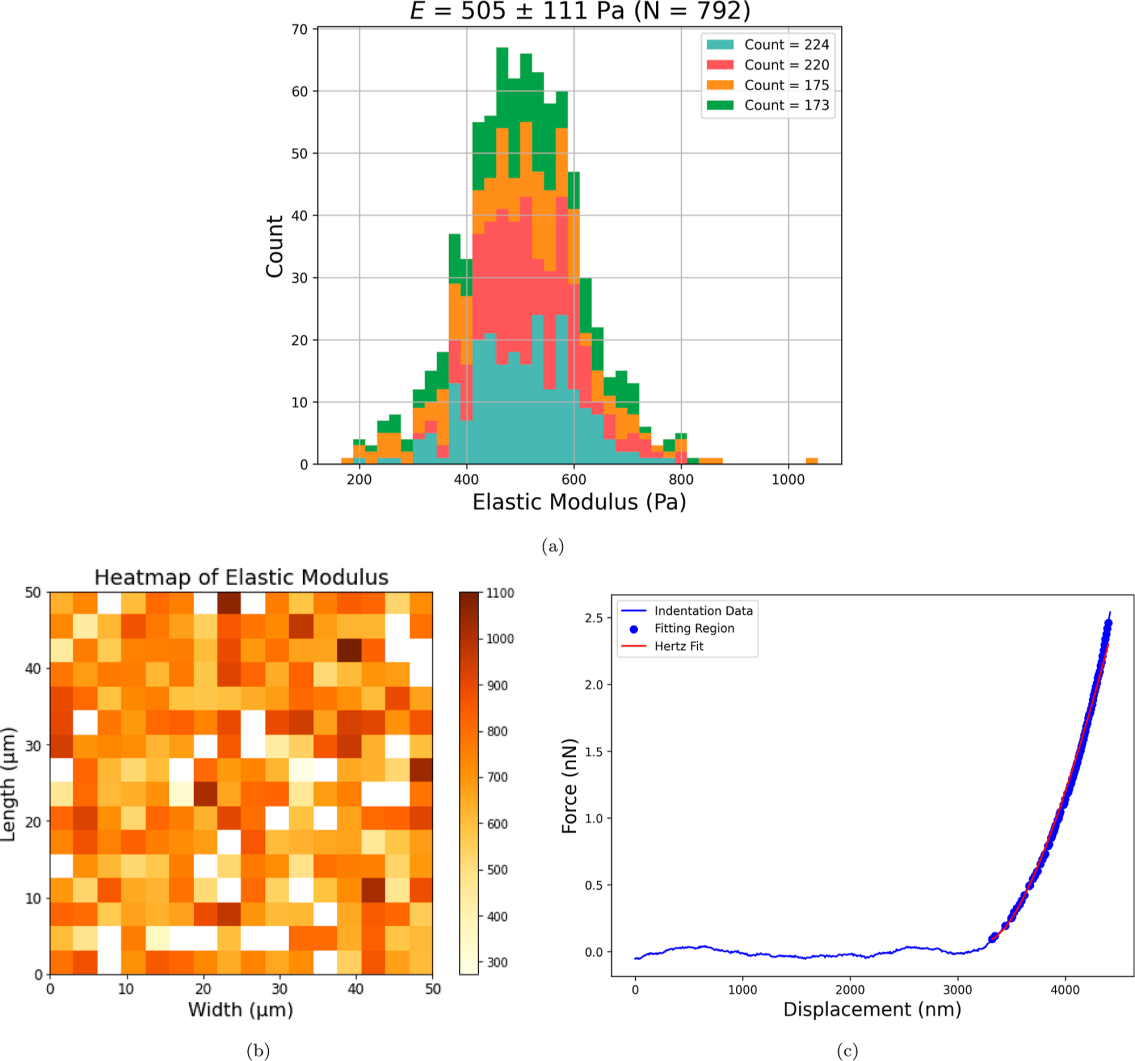
(a) Histogram of elastic modulus values, mean *E* = 505 ± 111 Pa. Range: 166–1055 Pa. Interquartile range: 140 Pa. *Count* corresponds to the number of indentation curves (out of 256) exhibiting compliance with Hertzian contact mechanics and is hence retained for analysis. (b) Elastic modulus measured in a 16 × grid across a 50 × 50 μm area of SPMK-*g*-PEEK submerged in PBS (count = 224). (c) Force–displacement indentation curve for a 10 μm-radius colloidal probe indenting SPMK-*g*-PEEK submerged in PBS, showing the region which is compliant with Hertzian contact fitting for calculating elastic modulus.

The elastic moduli of the swollen polyelectrolyte interfaces submerged in PBS for SPMK-*g*-PEEK were determined using AFM force mapping to be *E* = 505 with a standard deviation of ±111 Pa. This value indicates variability in the mechanical properties, with the observed range spanning from 166 to 1055 Pa, as depicted in Figure 4a. This analysis was based on 1024 indentation measurements conducted across two samples of SPMK-*g*-PEEK. Specifically, Figure 4b shows a representative 50 × 50 μm area, where the elastic moduli were assessed in a 16 × 16 grid. During the evaluation process, any force–displacement curves that either did not align with Hertzian contact mechanics or demonstrated significant error were excluded. Consequently, a total of 792 modulus measurements were retained for analysis. A representative force–displacement indentation curve is presented in Figure 4c, indicating that indentation depths of 1 μm consistently resulted in forces below 5 nN. Moreover, the curves did not adhere to Hertzian contact theory at forces approximately lower than 0.5 nN. The accurate determination of surface contact for soft materials poses a significant challenge, requiring the mathematical delineation of the indentation range that accurately fits the Hertz model (Figure 4c).^69,71^

### SPMK and PEEK Speed Sweep Analysis

**Figure 5:**
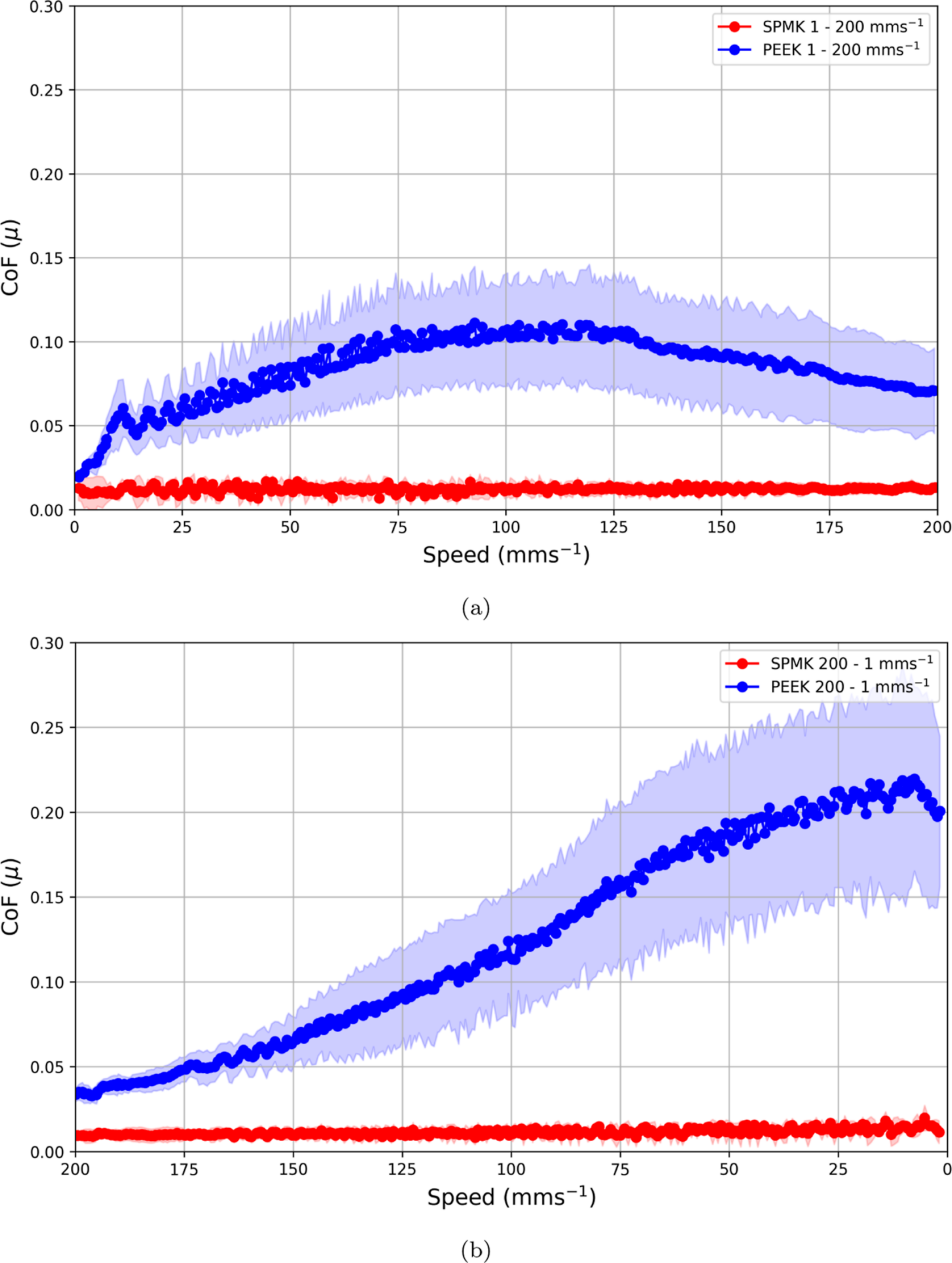
SPMK-*g*-PEEK (*N* = 3) and PEEK (*N* = 3) disc versus flat ⌀ 4 mm SCA cartilage plug for (a) increasing speed sweep from 1 to 200 mm/s and (b) decreasing speed sweep from 200 to 1 mm/s.

Figure 5a,b shows the CoF evolution for SCA cartilage against SPMK-*g*-PEEK and unfunctionalized PEEK during sweeps of increasing speed 1–200 mm/s and decreasing speed 200–1 mm/s, respectively. In both scenarios, SPMK-*g*-PEEK demonstrated a remarkable stability of CoF, exhibiting minimal variation with a mean CoF of μ = 0.012 ± 0.002 and μ = 0.011 ± 0.002 for the decreasing and increasing speed sweeps, respectively. Conversely, the CoF response of unfunctionalized PEEK exhibited significant variation dependent on the speed sweep direction. For the increasing speed case, CoF rises steadily up to a maximum μ = 0.11 ± 0.036 and begins to reduce at speeds above 120 mm/s to a final CoF of μ = 0.071 ± 0.025. In contrast, for the decreasing speed sweep, the CoF increased steadily, starting from μ = 0.034 ± 0.004 at a sliding speed of 200 mm/s and reaching a peak of μ = 0.22 ± 0.068 at 5 mm/s, before exhibiting a slight decrease when the sliding speed further reduced to 1 mm/s.

### Strain Recovery and Tribological Rehydration

**Figure 6:**
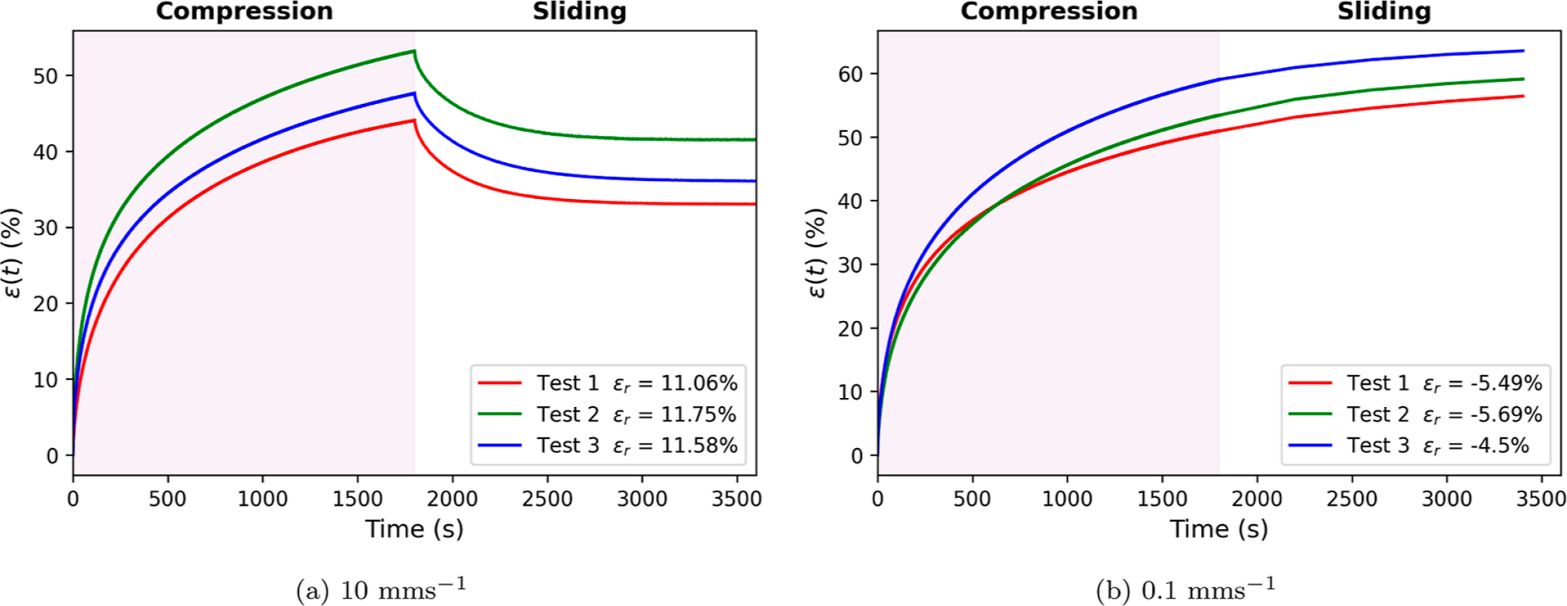
Strain evolution during the compression and sliding phases under an applied load of 90 N of ⌀ 7.2 mm SCA cartilage sliding against SPMK-*g*-PEEK at (a) 10 and (b) 0.1 mm/s.

The representative strain datum [ε(*t*)] for the 90 N rehydration cycles is shown in Figure 6 for the 10 mm/s (Figure 6a) and 0.1 mm/s (Figure 6b) conditions, demonstrating ε_r_ ∼ 11% and ε_r_ ∼ −5%, respectively, during the sliding phase. During the first 30 min of unconfined compression, the cartilage interstitial fluid exuded at a decaying rate toward a static equilibrium. Upon the onset of sliding, rehydration of the cartilage can occur, reducing the overall compression as fluid is reimbibed by the cartilage, which is clearly observed for the 10 mm/s condition (Figure 6a).

**Figure 7:**
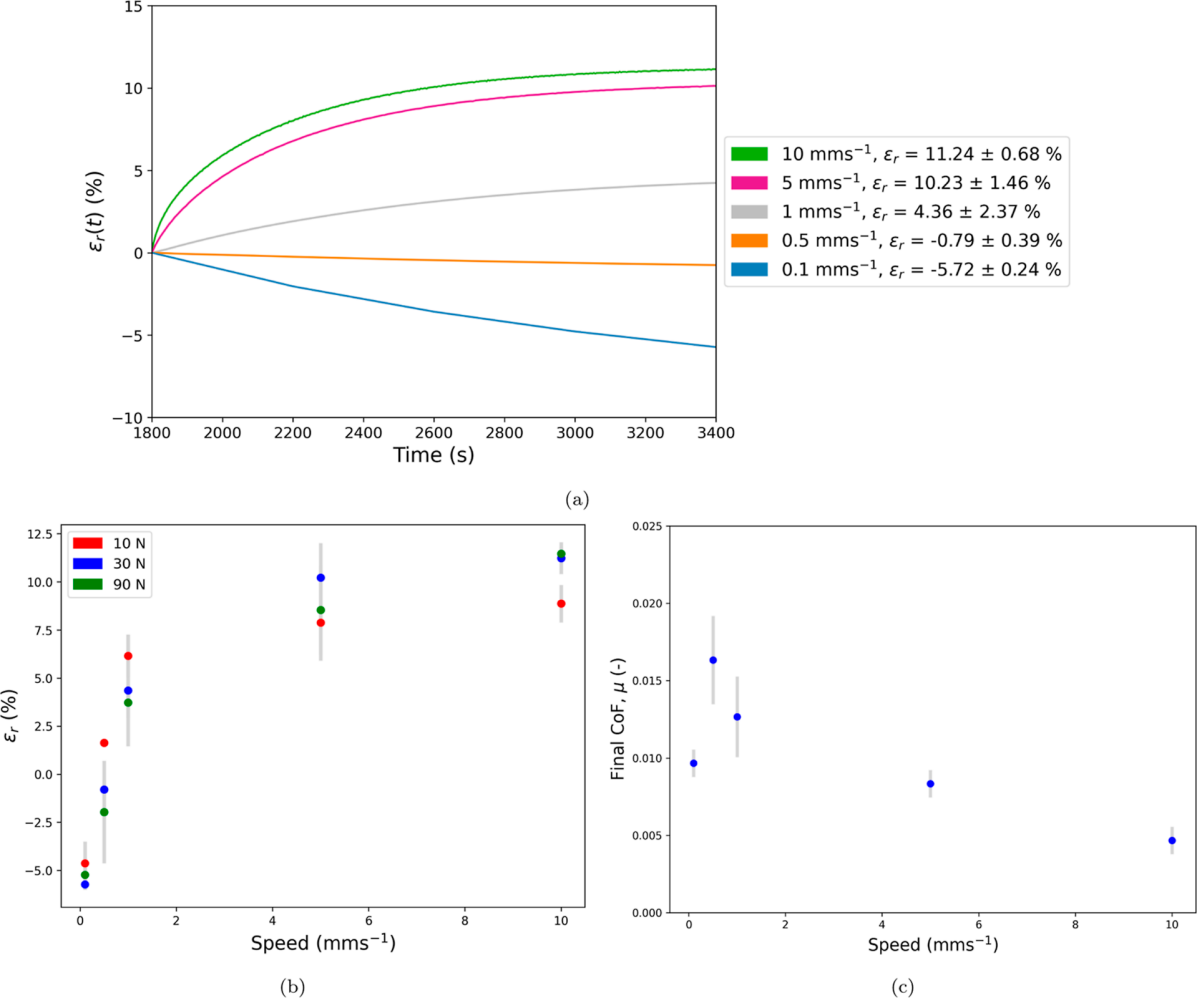
(a) Evolution of mean strain recovery [ε_r_(*t*)] for the 30 N load during the sliding phase of the rehydration cycle for all sliding speeds. Error bars are omitted for clarity. (b) Strain recovery (ε_r_) for all test conditions (Table 1) plotted for each speed condition with standard deviation error bars shown. (c) Mean CoF (μ) for the 30 N load during the sliding phase of the rehydration cycle for all sliding speeds with standard deviation error bars shown (Table 2).

The mean strain recovery (ε_r_, eq 3) was calculated for each speed and load condition with a sample size of *N* = 3 for each group. Figure 7a shows the evolution of the mean strain recovery [ε_r_(*t*)] throughout all sliding phases for the 30 N load condition. The overall mean strain recovery for each condition is plotted in Figure 7b and aggregated in Table 1 along with the mean strain at the end of the compression phase (ε_c_) for each load condition. Figure 7b shows that across all load conditions, an increase in strain recovery (ε_r_) was observed in correlation with increments in sliding speed. Notably, at a minimal speed of ν = 0.1 mm/s, the overall cartilage strain persistently augmented throughout the sliding phase, culminating in a negative recovery strain of approximately ε_r_ ∼ – 5% across all load conditions. Strain recovery due to tribological rehydration became pronounced at speeds exceeding ν = 1 mm/s, with the highest strain recovery for each condition being attained at the highest speed, ν = 10 mm/s. The analysis revealed that samples subjected to a 10 N load exhibited the least overall strain recovery at ν = 10 mm/s, with ε_r_ = 8.87 ± 0.79%, whereas the 30 and 90 N conditions demonstrated comparably higher maximum strain recoveries of ε_r_ = 11.24 ± 0.68% and ε_r_ = 11.46 ± 0.29%, respectively. The variability in cartilage strain recovery, indicated by a standard deviation range of ±0.11–2.37%, aligns with findings from prior studies exploring tribological rehydration-induced strain recovery in bovine cartilage.^76,77^ This observed strain error represents the inherent mechanical, poroviscoelastic, and thickness variations in cartilage samples harvested across a range of bovine specimens and patellofemoral locations.^78^

**Table 1:** Summary of Strain Recovery (ε_r_) Calculated across Each Specified and Load Condition with a Sample Size of *N* = 3 for Each Group^a^

**Load**	**Compression**	**Rehydration**, ε_r_ (%)				
*F*_*Z*_*(N)*	**ε_c_ (%)**	**0.1 mm/s**	**0.5 mm/s**	**1 mm/s**	**5 mm/s**	**10 mm/s**
10 N	26.1 ± 1.31	–4.63 ± 0.93	1.63 ± 0.11	6.15 ± 0.88	7.89 ± 1.62	8.87 ± 0.79
30 N	42.0 ± 1.39	–5.72 ± 0.24	–0.79 ± 0.39	4.36 ± 2.37	10.23 ± 1.46	11.24 ± 0.68
90 N	51.8 ± 2.91	–5.23 ± 0.52	–1.97 ± 2.18	3.73 ± 0.65	8.54 ± 1.87	11.46 ± 0.29

a Along with the cartilage strain at the end of the compression phase (ε_c_) for each load condition, with a sample size of *N* = 15 for each group.

The observation of strain recovery and subsequent cartilage rehydration increasing with sliding speed demonstrated consistency across all compressive stresses applied to the cartilage, quantified as ε_c_ = 26.1 ± 1.3, 42.0 ± 1.4, and 51.8 ± 3.0% for the 10, 30, and 90 N load conditions, respectively, as summarized in Table 1. Analyzing net strain recovery (ε_r_), Figure 7b illustrates that at lower sliding speeds of ν ≤ 1.0 mm/s, the 10 N condition facilitated a greater recovery of cartilage strain. A transition is evident at higher speeds, specifically ν ≥ 5.0 mm/s, where enhanced strain recovery is observed under the higher 30 and 90 N load conditions. This trend underscores the role of sliding speed, and subsequently hydrodynamic effects, in augmenting cartilage interstitial fluid recovery, evidenced by the increased cartilage strain recovery attributed to tribological rehydration facilitated by the polyelectrolyte SPMK interface.

Figure 7c shows the mean sliding phase CoF (μ) for the *F*_*Z*_ = 30 N load condition, with the data aggregated in Table 2. For all sliding speeds, SPMK-g-PEEK facilitated low friction with μ < 0.016 throughout the sliding cycle, aligning with previous research demonstrating the lubricating efficacy of polyelectrolyte–cartilage contacts.^31^ For increasing speeds between 0.5 and 10 mm/s, a decrease in CoF was observed from a maximum of μ = 0.016 ± 0.003 to a minimum of μ = 0.005 ± 0.001. This is commensurate with enhanced interstitial fluid pressurization evidenced by greater strain recovery across the increasing speed range (Figure 7a) and broadly aligns with the interstitial fluid pressurization hypothesis (eq 1).

**Table 2:** Summary of Mean CoF (μ) of the 30 N Load Condition at Speeds of 0.1–10 mm/s with a Sample Size of *N* = 3 for Each Group

**Load**	**CoF**, μ (–)				
*F*_*Z*_*(N)*	**0.1 mm/s**	**0.5 mm/s**	**1 mm/s**	**5 mm/s**	**10 mm/s**
30 N	0.010 ± 0.001	0.016 ± 0.003	0.013 ± 0.003	0.008 ± 0.001	0.005 ± 0.001

## Discussion

The interface between SPMK-*g*-PEEK and cartilage represents a significant advancement in the development of biomaterials aimed at mimicking the natural lubrication and mechanical properties of supramolecular complexes adsorbed on cartilage. This section explores the structural characterization of SPMK-*g*-PEEK, highlighting the swollen height, mechanical properties, and polyelectrolyte conformation designed to mimic synovial biopolyelectrolytes. Analysis of the tribological and strain recovery behavior of cartilage interfaced with SPMK-*g*-PEEK reveals sustained low friction akin to physiological levels and the ability to augment interstitial fluid load support, both necessary for maintaining the long-term function of cartilage. These findings are contextualized within the broader scope of cartilage lubrication models, culminating in a new hypothesis of polyelectrolyte-enhanced tribological rehydration.

### SPMK-*g*-PEEK Interface

The swollen height of SPMK measured in this study was ∼5 μm (Figure 3), an order of magnitude greater than the *R*_a_ ∼ 100 nm roughness of PEEK, which protects interfacing cartilage from hard asperity contact and hence provides a lubricious compliant interface. This is similar to the measured ∼1–10 μm MPC polymer brushes on steel^66^ and polyethylene^79^ substrates in the context of biomedical implants. Previous measurement of the dry height of the SPMK layer grafted to PEEK using FIB-SEM measured a 397 ± 47 nm-thick polyelectrolyte layer,^31^ meaning that the SPMK exhibits a swelling ratio of approximately 12×. The measured SPMK thickness demonstrates that swelling and compression of the polyelectrolyte is too small to contribute to the overall strain recovery of articular cartilage. Typical cartilage thickness was approximately 1200 μm which when considering strain recovery in the region of ε_r_ ∼ −5–12% corresponds to an approximate height change of 60–200 μm.

Nanomechanical analysis of the SPMK-*g*-PEEK demonstrated that the SPMK-*g*-PEEK surface submerged in PBS has a modulus of *E* = 505–111 Pa, exhibiting a highly compliant surface consistent with extended polyelectrolyte chains with a high fluid content.^71^ This reflects the highly compliant 1–20 μm-thick^22,24−26^ superficial macromolecular complex adsorbed on cartilage with a modulus of *E* = 9 ± 2 kPa.^26^ The SPMK surface moduli are markedly lower than those in previous literature exploiting biomedical applications of polyelectrolytes. AFM force mapping of brush-terminated hydrogels designed to mimic hydrophilic proteins native to corneal or synovial surfaces exhibits moduli of *E* ∼ 3–44 kPa.^71,80,81^ These are orders of magnitude lower than previously reported biomedical applications of MPC grafted to rough polyethylene (*R*_a_ = 650 μm) hip replacement implants with swollen MPC height ∼1400 nm thick and AFM nanomechanical studies measuring a variable modulus of *E* = 73 ± 72 kPa due to varying substrate effects.^79^

**Figure 8:**
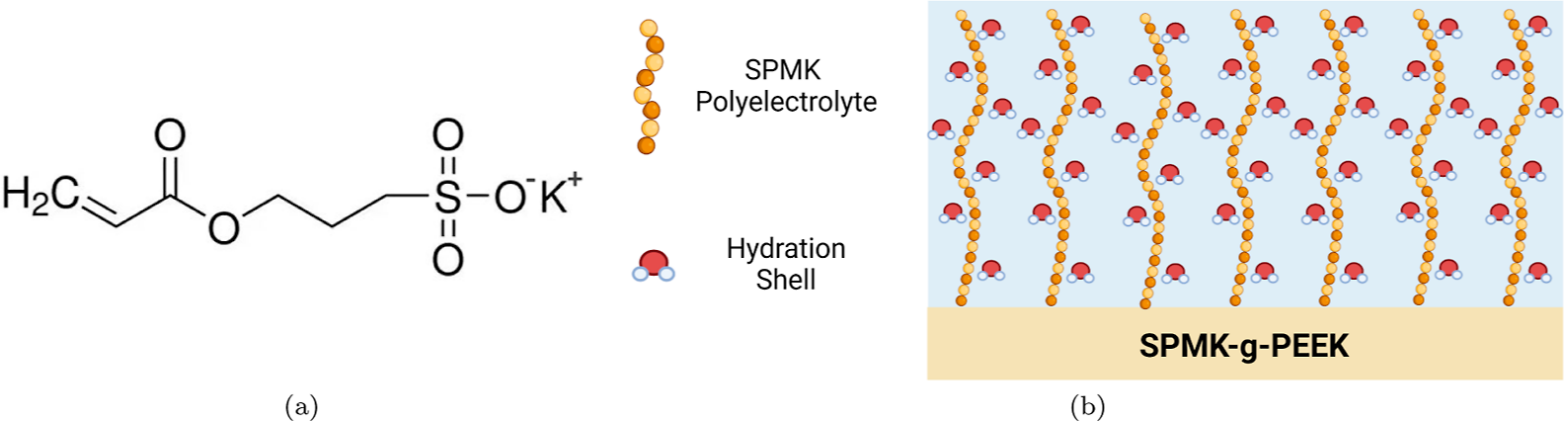
(a) SPMK monomer. (b) Polymer brush conformation of SPMK-*g*-PEEK showing the presence of bound hydration shells on the sulfonic acid groups.

The observed low moduli and ∼5 μm swollen height demonstrate that *grafting from* of the SPMK monomer (Figure 8a) onto PEEK substrates yields a dense end-tethered polymer surface (Figure 8b) enriched with sulfonic acid groups.^31,67^ The sulfonic acid groups possess hydrophilic and ionizable characteristics, enabling them to dissociate in aqueous environments leaving negatively charged sulfonate ions (SO_3_^–^) tethered to the polymer backbone, the same hydrophilic functional groups present on proteoglycans in synovial fluid.^2^ Electrostatic repulsion among the negatively charged SO_3_^–^ groups and osmotic pressure of hydrated counterions around the charged chains cause the polymer chains to extend away from the substrate and form a brush-like configuration.^2,47,82−84^ The highly hydrophilic sulfonate groups form tight hydration shells contributing to the solvation of the polymer brush supporting its extended formation and facilitating hydration lubrication.^28^ Such polymer brush structures can resist deformation under compressive loading due to the conformational entropy and exclude the volume effect of the hydrated SPMK polyelectrolyte causing a repulsive force under loading.^84,85^

### SPMK-*g*-PEEK–Cartilage Tribology

This study clearly demonstrates SPMK-*g*-PEEK’s tribological efficacy as a cartilage counterface under physiological loads of 0.75–2.21 MPa. Hydrated SPMK provides a highly lubricious surface capable of maintaining an invariably low CoF of μ ∼ 0.01 across a speed range of 1–200 mm/s and mechanism to augment cartilage strain recovery of up to ε ∼ 11.5%. Under constant loading, the rehydration cycle demonstrates that the recovery of cartilage interstitial fluid increases with sliding speed (Figure 7b), evidenced by the increasing strain (eq 1), highlighting the role of hydrodynamics in facilitating polyelectrolyte-enhanced tribological rehydration. At higher sliding speeds, greater strain recovery appears to facilitate lower friction coefficients, as shown by the CoF trend analysis for *F*_*Z*_ = 30 N (Figure 7c) which exhibits the lowest μ = 0.005 ± 0.001 at the high speed condition of ν = 10 mm/s along with the greatest strain recovery ε_r_ = 11.24 ± 0.68%. Maintenance of low friction and the reduction of cartilage strain position SPMK-*g*-PEEK as a promising material for maintaining cartilage health. Effective rehydration of articular cartilage is important to maintain cell viability^86^ and provide fluid flow for solute transport and removal of metabolic waste from the tissue.^87,88^ Furthermore, both effective rehydration and high lubricity are crucial for shielding the collagen matrix from high shear and normal forces to prevent wear.^4,89^

Speed sweep analysis (Figure 5) of unfunctionalized PEEK is representative of the current understanding of SCA models, exhibiting the lowest CoF μ ∼ 0.02–0.04 at the start of sliding and hence at the point of the minimum strain, irrespective of the 1 mm/s and 200 mm/s starting speeds. For PEEK, the increasing 1–200 mm/s speed condition shows a peak CoF μ ∼ 0.11 at speeds of 120 mm/s recovering slightly at higher speeds likely due the onset of a soft-EHL regime,^90^ whereas for the decreasing speed condition, the peak CoF μ ∼ 0.22 occurred at the end of testing corresponding to maximum temporal strain. SPMK-*g*-PEEK demonstrated invariably low CoF for both increasing and decreasing speed sweeps with μ < 0.012 in both scenarios, maintaining high lubricity that is unaffected by variations in loading time (*i.e.*, contact strain), speed, or lubrication regime. Highlighting that for aqueous lubrication systems with the ability to hold water at the surface, friction cannot necessarily be associated with a change in the lubrication regime.^3^ Similar speed-independent CoF (μ ∼ 0.02, ν = 0.1–50 mm/s) has been shown for the aqueous lubrication of hydrophilic poly(ethylene glycol) brushes^91,92^ and brush-terminated hydrogels in self-mated Gemini contacts.^93,94^ This has been attributed to an elastoviscous regime, where the extended polymer chains can influence the interfacial viscosity which has a net smoothing effect to damp frictional transitions between boundary and fluid film lubrication regimes.^94^ Furthermore, at low speeds in confined interfaces, high polyelectrolyte concentration can increase effective viscosity and produce substantially higher film thickness than expected for conventional elastohydrodynamic theory at low speeds, giving rise to a low speed (≥0.1 mm/s) onset of fluid film lubrication.^60,62,72,91^ The lubricating efficacy of SPMK-*g*-PEEK is attributed to the confined polyelectrolyte behaving as a viscous lubricant to produce lubricating fluid films at low speeds.^60,62,91^ When considering the high roughness of cartilage (*R*_a_ ∼ 500 nm^95^), it is likely that this is a localized phenomenon in regions of cartilage asperity contact. Notably, CoF decreases with increasing speed in tandem with an increasing strain recovery during longer-term testing of the rehydration cycle (Figure 7c), demonstrating that polyelectrolyte-enhanced lubricating fluid films exhibit shear-thinning behavior^96^ and can promote low friction synergistically with maintenance of interstitial fluid pressurization.

Early cadaveric hip pendulum studies to simulate gait show that for human joints, CoF was typically between a range of μ ∼ 0.01 and 0.04^97−99^ and are corroborated by recent benchtop cartilage–cartilage tribology studies showing CoF as low as μ ∼ 0.001–0.015.^4,39^ However, the current state of research applies a reductionist approach to discern between three modes of MCA, cSCA, and SCA tribological rehydration.^14,100^ Studies using a hard impermeable counterface (*i.e.*, glass, PEEK) show that during sustained sliding MCA and cSCA cartilage conditions, friction can remain consistently as low as μ ∼ 0.03 as a result of maintaining low cartilage strain and high interstitial fluid pressurization (eq 1).^39,40^ MCA cartilage on glass exhibits low CoF values of 0.01–0.07 between speed ranges of 0.05 and 4.5 mm/s, maintaining a fluid load support of  ∼ 0.85–0.9.^38,39,101^ Tribological rehydration of cSCA cartilage is shown to only occur at speeds above 30 mm/s when hydrodynamic pressures are sufficient to promote interstitial fluid recovery, demonstrating low CoF μ ∼ 0.01–0.03 at high speeds of 80 mm/s ( 0.9) and high CoF values of μ ∼ 0.1–0.4 at lower speeds of 1–20 mm/s below the speed threshold for effective interstitial fluid recovery.^40,76,102^ SCA cartilage sliding experiments are analogous to cartilage in unconfined compression, exhibiting no evidence of fluid imbibition to compete with the interstitial fluid efflux during loading.^31,39^ At low speeds of 1 mm/s, SCA cartilage exhibits a CoF of μ ∼ 0.19,^39^ increasing up to μ ∼ 0.3–0.5 at speeds of 80 mm/s.^40,102^ The CoF observed in the presented rehydration cycles consistently remains low (μ ∼ 0.01), reflecting the physiological friction coefficients present in synovial joints.^4,39,97−99^ This study’s observation of SCA cartilage maintaining low friction at low velocities (ν = 0.1–10 mm/s) diverges from extant cartilage rehydration frameworks, demonstrating tribological and rehydration dynamics akin to those elucidated in MCA and cSCA cartilage models,^38−40,101,102^ which highlights an unexplored avenue of tribological rehydration facilitated by polyelectrolyte boundary lubrication interfaces, mirroring the configuration of endogenous superficial macromolecular complexes, yet neglected by prevailing MCA and cSCA paradigms.

The mean strain recovery (ε_r_) for all speed and load conditions (7b) shows that as speed increases, the recovered strain and subsequently the interstitial fluid pressurization increase. Compared to cSCA tribological rehydration which only occurs at speeds of above 30 mm/s,^40,76,102^ the polyelectrolyte-enhanced tribological rehydration demonstrates a net recovery of strain even in low speed conditions of ν = 0.1–0.5 mm/s. This is hypothesized to be underpinned by the low speed affinity of polyelectrolytes for enhanced fluid film formation^60,62,91^ promoting hydrodynamic pressurization and restoration of interstitial fluid. Strain recovery (ε_r_) becomes asymptotic in all speed conditions, corroborating similar findings that an equilibrium is reached between the interfacial and interstitial pressure fields.^40,76,77^ Maximum strain recovery (ε_r_) observed at ν = 10 mm/s is lower for the *F*_*Z*_ = 10 N condition (ε_r_ = 8.76 ± 0.79%) compared to the higher load conditions of *F*_*Z*_ = 30 and 90 N which exhibit ε_r_ ∼ 11%, which intuitively demonstrate that at greater loads, greater fluid pressurization occurs, leading to greater strain recovery.^72^ A transition around ν = 1 mm/s is observed; below this threshold, greater strain recovery occurs for the 10 N load, whereas above this transition, the strain recovery rate for 30 and 90 N load conditions becomes greater than that at *F*_*Z*_ = 10 N. The permeability of cartilage is inversely proportional to compressive strain,^103^ which for low loads will mean that the net fluid flow of cartilage can occur at a greater rate following Darcy’s law.^104^. The speed transition observed in Figure 7b corroborates the previous hypothesis of greater fluid pressurization at higher speeds, yielding a greater rate of interstitial fluid recovery toward equilibrium.

### Hypothesis of Polyelectrolyte-Enhanced Tribological Rehydration

Coupling of hydrodynamic and interstitial fluid pressure fields has been developed for explaining tribological rehydration of cSCA cartilage.^40,41^ Specifically, this has been undertaken as a percolation-based approach to mixed lubrication of cartilage treated as a porous material.^41,105^ Within this interface, modeling of hydrodynamic forces induced by a wedge effect hypothesizes fluid pressure peaks at the contact inlet, facilitating interstitial fluid recovery. Additionally, rehydration within the loaded contact zone occurs as fluid trapped at asperity contacts becomes pressurized, forming localized rehydration channels.^41^ This process leverages the intrinsic roughness of cartilage to create percolating channels. Compliant tribological systems such as cartilage have been shown to flatten at moderate pressures, which is advantageous for reducing friction in hydrodynamic lubrication.^96,106^ The percolation approach models cartilage as a material with multiple roughness scales, postulating that the microroughness of cartilage must be present at the contact interface to maintain lubrication and facilitate rehydration.^105^

Understanding the lubrication of SPMK-*g*-PEEK–cartilage interfaces necessitates an adaptive multimode lubrication model^107^ due to the dominating role of interstitial fluid pressurization in supporting the majority of applied load,^19,21,23^ along with the boundary lubrication expected to occur when pressurization subsides and cartilage contact occurs.^3,19^ To the authors’ knowledge, there have been no theoretical or experimental studies on the role of biological or synthetic polyelectrolytes for cartilage rehydration. Experimental^108,109^ and modeling^22^ approaches have explored the role of the presence of polyelectrolytes on cartilage, showing that the adsorbed superficial zone acts as a low permeability barrier, providing flow resistance to sustain cartilage interstitial pressure which is congruent with our initial published studies.^31^ However, this does not explain the net strain recovery observed at the onset of sliding (Figure 7b). Any potential cushioning effect of the ∼5 μm-thick low modulus SPMK interface providing rehydration through passive swelling is contradicted by previous studies that have shown no notable reduction in cartilage strain when comparing PEEK and SPMK-*g*-PEEK^31^ and passive swelling rates being slower than tribological rehydration^110^ suggesting a reduced load-speed dependency than observed (Figure 7b). Instead, cartilage rehydration is an active process onset by sliding (Figure 6a) that competes with fluid exudation under loading.^40^

**Figure 9:**
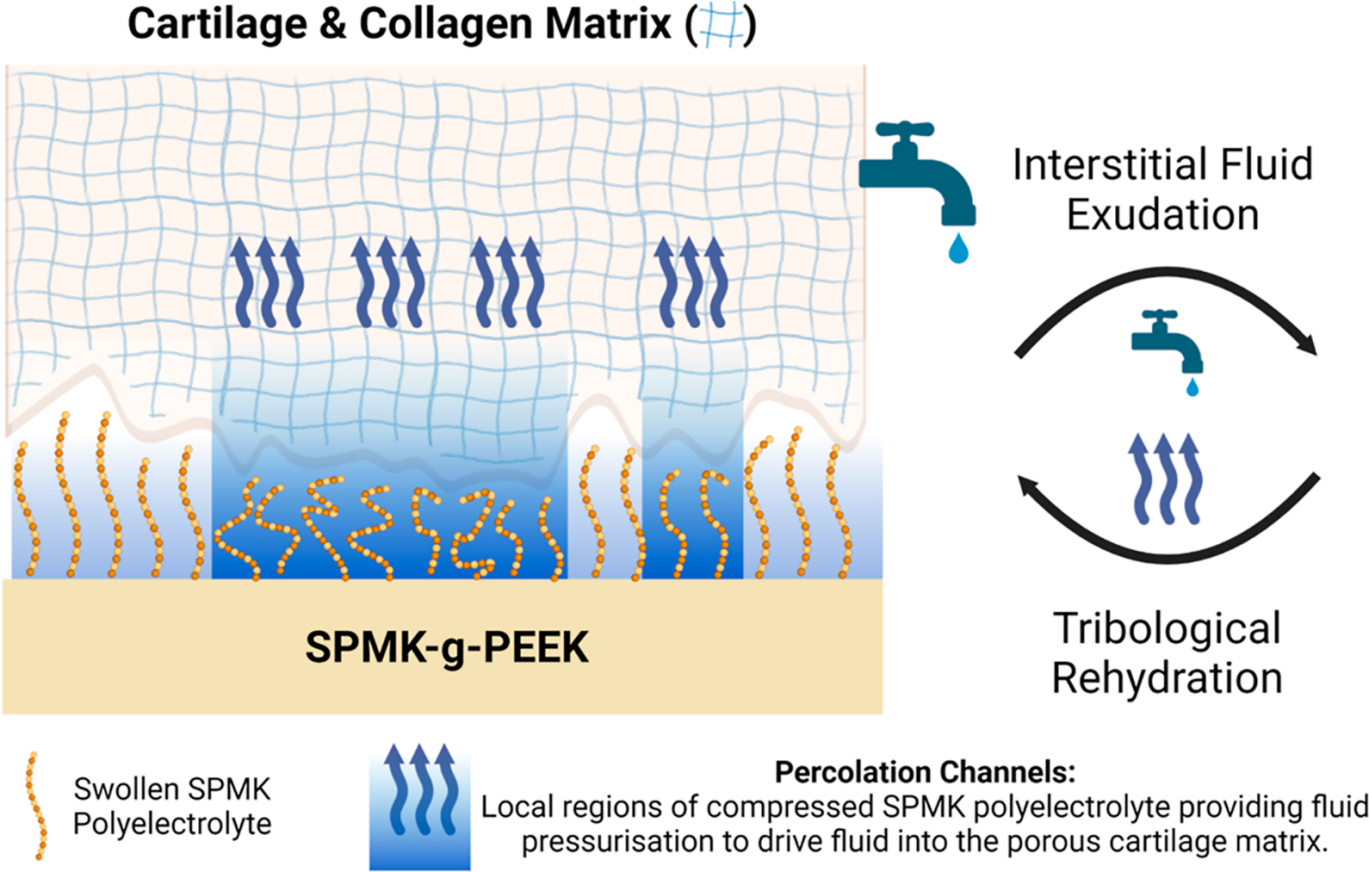
Hypothesized mechanism of polyelectrolyte-enhanced tribological rehydration. This process is conjected to occur through localized compression of the SPMK polyelectrolyte at cartilage asperities during sliding, which generates pressurized fluid regions within percolation channels to facilitate cartilage rehydration. Low friction is expected to be maintained by a polyelectrolyte-enhanced elastohydrodynamic fluid film and the highly hydrated SPMK boundary interface.

Figure 9 presents a hypothetical mechanism of polyelectrolyte-enhanced tribological rehydration; a similar percolation approach is considered by assuming that at the microscale level, cartilage still exhibits some roughness,^41^ giving rise to localized regions of compressed polyelectrolyte at cartilage asperities. Upon the onset of sliding, there will be a lubricant flow incurred, giving rise to a viscous fluid film enhanced by polyelectrolyte elastohydrodynamic lubrication.^60^ Compression of the hydrated SPMK polyelectrolyte will reduce the volume available for water molecules, and compounded by the increased relaxation times of polymer brushes in compression,^46^ produce pressurized regions of water which overcome the cartilage interstitial fluid pressure and facilitate rehydration.^3,47^ Increased strain recovery at greater speeds is expected to be a convolution of enhanced fluid film formation due to the polyelectrolyte,^60,62,91^ resulting in a greater quantity of fluid at the interface, along with a greater percolating flux exposing the cartilage asperities to more polyelectrolyte per unit time.^41^

Current cartilage models posit that interstitial fluid pressurization is the dominant mechanism to maintain low CoF in cartilage and overlooks the role of biological polyelectrolytes.^18,39−41^ The holistic role of lubricating biopolyelectrolytes found in synovial fluid remains a contentious issue in biotribology research. Addition of synovial fluid into MCA and cSCA contacts shows no statistically significant reduction in friction or augmentation of interstitial fluid pressure.^39,76^ In contrast, a cornucopia of tribological research asserts the lubrication benefits of synovial fluid macromolecular complexes demonstrated at the nanoscale,^2−4^ in SCA and MCA cartilage contacts,^39,111−114^ and in whole joint models.^115,116^ However, these studies do not address the potential mechanisms by which these complexes might contribute to the rehydration of cartilage. The demonstration of polyelectrolyte-enhanced tribological rehydration in this study benefits from a precisely controlled chemical composition of direct attachment of SPMK to the substrate, whereas the *in vivo* adsorbed macromolecular complex relies on electrostatic interaction with the negatively charged cartilage surface to remain attached,^3,23^ which inevitably becomes challenging to maintain within the contact area during *in vitro* tribology studies particularly during testing of unmatched cartilage contacts.^39,76^ Engineering of surface-grafted polyelectrolytes provides a compelling solution not only to emulate the *in vivo* performance of cartilage but also as a versatile model for understanding the tribological phenomena of natural synovial lubrication.

### Future Work and Limitations

CryoSEM offers only an approximate measure of the swollen height of the SPMK polyelectrolyte while illustrating the distribution of the sulfonic acid groups. However, the spatial resolution of EDX, limited to 1 μm,^75^ necessitates additional methods such as ellipsometry^46^ for precise measurement of the swollen height at the SPMK-*g*-PEEK interface. Initial efforts to gauge the thickness of SPMK under hydrated conditions have proved challenging. This difficulty is largely due to the high water content and low relative polymer content, which result in a minimal change in polarization signals. Consequently, this measurement is still under active investigation.

No discussion in this study has been made regarding the potential interaction between the SPMK polyelectrolyte and any adsorbed superficial macromolecular complex present on the cartilage samples. Previous cartilage studies have shown that extensive washing with PBS can diminish or remove the superficial layer,^26,117^ and consideration of how the surface would become degraded through exposure to PBS during cutting, storage, or pretest free swelling in PBS was unaccounted.

Discerning between the impact on friction due to the SPMK-*g*-PEEK interface, which provides an unabating highly lubricious sliding interface, and interstitial fluid pressurization remains uncertain. The decrease in friction observed in this study for increased states of cartilage rehydration (Figure 7c) suggests that a synergy between cSCA and polyelectrolyte-induced tribological rehydration is possible. Future studies should explore the behavior of SPMK-*g*-PEEK using a cSCA cartilage model.

The cartilage–SPMK-*g*-PEEK interface presented in this study presents a challenging numerical modeling problem, requiring interfacing the interstitial fluid flow and strain of cartilage with the local fluid pressurization of compressed polyelectrolyte chains, necessitating the combination of a molecular dynamics problem^118^ coupled with a poroviscoelastic cartilage model which accounts for the multimode lubrication regime and strain-dependent cartilage topography.^13^

## Conclusions

Hydrophilic SPMK polymer brush surfaces tethered to PEEK substrates have been developed as an advanced biomaterial to interface directly with cartilage and support native biotribology. These surfaces draw inspiration from the macromolecular constituents of synovial fluid, aiming to replicate its lubricating properties. The development of SPMK-*g*-PEEK surfaces, featuring a hydrated tethered layer approximately 5 μm thick, facilitates low friction coefficients (μ ∼ 0.01) over a broad speed range (0.1–200 mm/s) under physiological loading conditions (0.75–1.2 MPa). A pivotal finding of this study is the discovery of a novel polyelectrolyte-enhanced tribological rehydration mechanism capable of recovering cartilage interstitial fluid under loads ranging from 0.25 to 2.21 MPa. This recovery is attributed to the synergistic effects of fluid confinement within the contact gap and the enhanced elastohydrodynamic performance of the polymer brushes.

Going beyond prevailing theories that attribute cartilage lubrication to interstitial fluid pressurization and tribological rehydration through conformal geometries, our findings demonstrate that physiological friction coefficients of SPMK-*g*-PEEK interfaced with cartilage can occur independently of interstitial fluid recovery and pressurization. This discovery challenges existing paradigms and suggests a novel mechanism of lubrication that does not solely rely on the established models of interstitial fluid pressurization. The implications of this research extend beyond the specific interactions of SPMK-*g*-PEEK with cartilage, offering a broader understanding of synovial joint lubrication. By synthesizing materials that replicate the superficial macromolecular complex of cartilage, we have elucidated a new mechanism for the regulation of cartilage interstitial fluid. This advances our understanding of joint lubrication and opens new avenues for the development of joint replacement materials that more closely mimic the natural function of cartilage.

## Data Availability

The data associated with this paper are openly available from the Mendeley Data repository. Available: Elkington, Rob (2024), “Brushing Up On Cartilage Lubrication: Polyelectrolyte Enhanced Tribological Rehydration”, Mendeley Data, V1, doi: 10.17632/xmh2tyymdx.1.

## Supporting Information Available

NPFlex surface roughness (*R*_a_) measurements of the dry PEEK and SPMK-*g*-PEEK along with height maps (PDF)

## Notes

Inclusion in Langmuir: Nicholas Spencer, who is honored with this virtual issue of Langmuir, has made important contributions into understanding the aqueous lubrication of polymer brush systems and their inherent similarity to the lubricating biopolyelectrolytes present in synovial biotribology.^3,44^ Creative Commons: For the purpose of open access, the author has applied a Creative Commons Attribution (CC BY) license to any Author-Accepted Manuscript version arising from this submission.
