# Supplementary material for: Brushing Up on Cartilage Lubrication: Polyelectrolyte-Enhanced Tribological Rehydration
Source: Langmuir. 2024 May 7;40(20):10648–62. doi: 10.1021/acs.langmuir.4c00598 (PMC11112737; doi:10.1021/acs.langmuir.4c00598)
Supplement: Supplementary file 1 — la4c00598_si_002.pdf [file la4c00598_si_002.pdf]

Supporting Information:
Brushing Up On Cartilage Lubrication: Polyelectrolyte Enhanced Tribological Rehydration

Robert J Elkington[a,*], Richard M Hall[b], Andrew Robert Beadling[a], Hemant Pandit[c], Michael G Bryant[b]

[a]*Institute of Functional Surfaces, Mechanical Engineering, University of Leeds, Leeds, LS2 9JT, Yorkshire, UK*
[b]*School of Engineering, College of Engineering and Physical Sciences, University of Birmingham, Birmingham, B15 2TT, West Midlands, UK*
[c]*Leeds Institute of Rheumatic and Musculoskeletal Medicine, Chapel Allerton Hospital, Chapeltown Road, Leeds, LS7 4SA, Yorkshire, UK*

Keywords: Polymer Brush, Cartilage, Cartilage Repair, Tribological Rehydration, BioTribology, Polyectrolye Surfaces

S1. NPFlex Roughness Measurements

A NPFLEX (Bruker, USA) optical interferometer was used to measure the surface roughness of the polished unfunctionalised PEEK and SPMK-g-PEEK samples using a non-contact vertical scanning interferometry (VSI) method, analysing surface reflections to create interference fringes at a 50× optical magnification. Three different 250 × 250 μm areas of each sample were scanned using a high intensity monochromatic green light to enhance reflection and minimise data loss. Optical profilometry data was processed using Bruker Vision64 software to calculate the mean arithmetic roughness (R_a) for each sample area.

Table S1 summarises the average roughness for three (N = 3) areas of both dry PEEK and SPMK-g-PEEK. Surface roughness of the unfunctionalised PEEK measured a mean roughness of $R_a = 101 \pm 9.8$ nm (N = 3), and mean roughness of the SPMK-g-PEEK measured $R_a = 304 \pm 10.9$ nm (N = 3).

Table S1: Average roughness (R_a) of three (N = 3) 250 × 250 μm areas of dry PEEK and SPMK-g-PEEK along with the calculated mean and one standard deviation (S.D.) for each sample.

	Average R_a (μm), (N = 3)			Mean ± S.D. (μm)
PEEK	92	99	115	101 ± 9.8
SPMK-g-PEEK	289	310	314	304 ± 10.9

Representative roughness measurements for dry unfunctionalised PEEK and SPMK-g-PEEK are shown in Figure S1.

*Corresponding author; Robert J Elkington, Email: *mnrje@leeds.ac.uk*

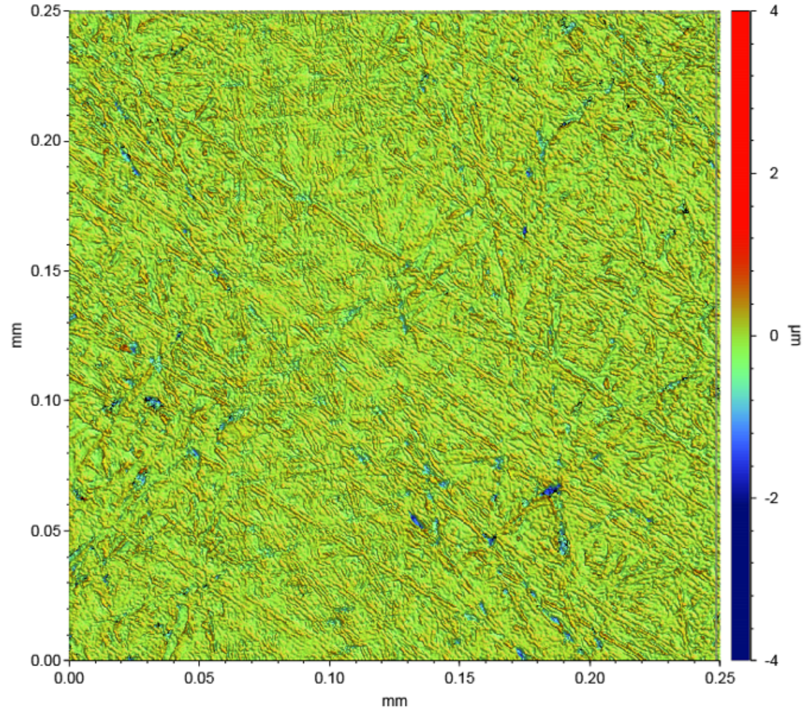

(a)

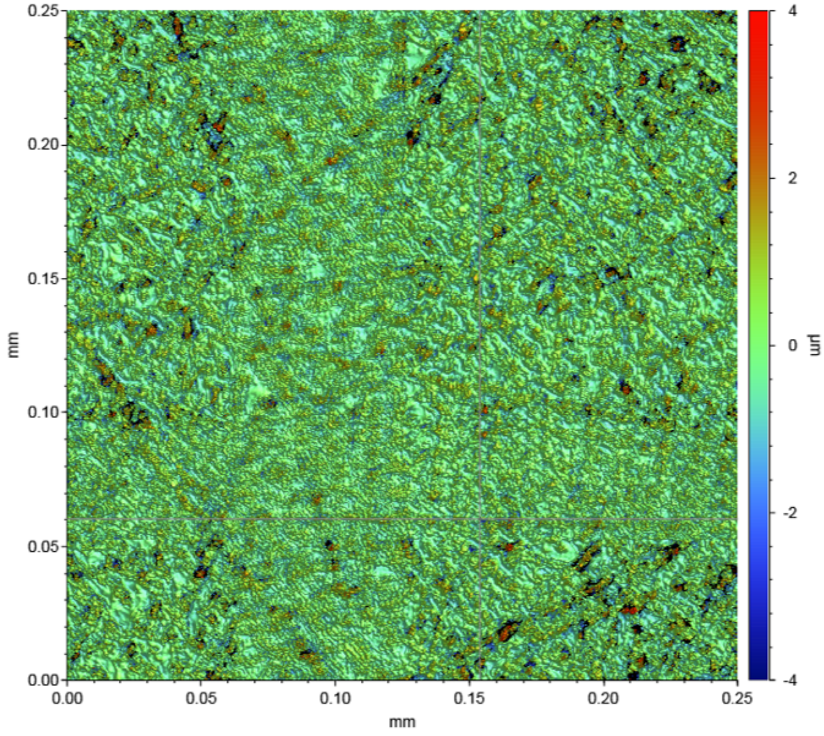

(b)

Figure S1: Representative height maps measuring the surface profile heights of S1a PEEK (R_a = 101 ± 9.8 nm (N = 3)) and S1b SPMK-g-PEEK (R_a = 304 ± 10.4 nm (N = 3)).
